# Surgical anatomy of the pectineal ligament during pectopexy surgery: The relevance to the major vascular structures

**DOI:** 10.4274/tjod.galenos.2020.21284

**Published:** 2020-04-06

**Authors:** Çiğdem Pulatoğlu, Ozan Doğan, Mahmut Sabri Medisoğlu, Murat Yassa, Aşkı Ellibeş Kaya, İlker Selçuk, Rahime Nida Bayık

**Affiliations:** 1 İstinye University Hospital Gaziosmanpaşa Medical Park, Clinic of Obstetrics and Gynecology, İstanbul, Turkey; 2 Private Clinic, Clinic of Obstetrics and Gynecology, İstanbul, Turkey; 3 Gölcük District Health Directorate, Department of Anatomy, Kocaeli, Turkey; 4 Sancaktepe Şehit Professor İlhan Varank Training and Research Hospital, Clinic of Obstetrics and Gynecology, İstanbul, Turkey; 5 Düzce University Hospital, Clinic of Obstetrics and Gynecology, Düzce, Turkey; 6 University of Health Sciences Turkey, Ankara Bilkent City Hospital, Clinic of Gynecologic Oncology/Hacettepe University Faculty of Medicine, Department of Anatomy, Ankara, Turkey; 7 University of Health Sciences Turkey, Göztepe Training and Research Hospital, Clinic of Obstetrics and Gynecology, İstanbul, Turkey

**Keywords:** Cadaveric study, major vessels, pectineal ligament, pectopexy, pelvic organ prolapse, vascular anatomy

## Abstract

**Objective:**

During pectopexy surgery, the prolapsed uterus or the vaginal apex is fixed to the pectineal ligament. The anatomic structures found in the lateral part of the prevesical and paravaginal space above the obturator fossa, raise the importance of the surgical steps required to prevent complications. This study was conducted to evaluate the proximity of vascular structures to the pectineal ligament.

**Materials and Methods:**

The distances between the surgical suturing area during pectopexy surgery and the external iliac vein, pubic anastomotic vessel (corona mortis) and obturator canal were measured bilaterally in seven fresh female cadavers.

**Results:**

The total length of the pectineal ligament was 5.9±0.76 cm on the left and 6.5±1.14 cm on the right side; the midpoint of the pectineal ligament was 2.8±0.52 cm on the left and 3.6±0.47 cm on the right side. From the midpoint of the left pectineal ligament, the mean distance to the left external iliac vein was 1.04±0.23 cm, to the left corona mortis it was 2.15±0.48 cm, and to the left obturator canal it was 3.12±0.95 cm. From the midpoint of the right pectineal ligament, the mean distance to the right external iliac vein was 1.25±0.43 cm, to the right corona mortis it was 2.37±0.63 cm, and to the right obturator canal it was 3.57±0.93 cm.

**Conclusion:**

The anatomic findings of the study confirmed that the pectineal ligament was in close association with main vessels. The external iliac vein was measured as the closest anatomic structure to the pectineal ligament. Surgeons must be careful to minimize life-threatening complications because of the proximity of the pectineal ligament to main vessels.

**PRECIS:** The findings of the study confirmed that the pectineal ligament was in close association with main vessels, the external iliac vein was measured as the closest structure.

## Introduction

There are many ligaments and delicate structures in the pelvis to which attention must be paid in surgical procedures. The importance of these anatomic structures emerges with newly documented surgical procedures and clinical anatomy studies. The pectineal ligament is one of the key anatomic structures in the pelvis, and is used in both abdominal and gynecologic surgeries. During procedures such as inguinal hernia repair, pelvic organ prolapse (POP), and stress urinary incontinence (SUI), the pectineal ligament is used as a surgical anchoring point^(1)^. Detailed anatomic knowledge of the pectineal ligament and relevant anatomic landmarks improves surgical anatomy practice and facilitates surgery.

Pectopexy surgery, which is performed for POP, was first described by Banerjee and Noé^(2)^. During this surgical procedure, the prolapsed uterus or the vaginal apex is fixed to the pectineal ligament with a mesh. The anatomic structures found in this area, the lateral part of the prevesical and paravaginal space above the obturator fossa, raise the importance of surgical steps performed during the procedure to prevent probable complications^(3)^. This cadaveric study was conducted to evaluate the proximity of anatomic landmarks and vascular structures to the pectineal ligament related with the pectopexy surgery.

### Clinical anatomy of the pectineal ligament

The pectineal ligament, which is also known as Cooper’s ligament, was first described by Sir Astley Cooper as the ligamentous extension lying over the iliopectineal line^(4)^. Complete knowledge of the inguinal region is crucial to understand the anatomy of the pectineal ligament. The inguinal ligament, which is the anterior border of the femoral canal, lies between the anterior superior iliac spine and pubic tubercle. The posterolateral reflection of this ligament from the pubic tubercle forms the lacunar ligament, which is the medial border of the femoral canal. From the pectineal attachment of the lacunar ligament, the fibrous connective tissue called the pectineal ligament, which is a ligamentous extension, lies laterally through the iliopectineal line below the superior pubic ramus. The medial part of pectineal ligament close to the pubic tubercle is the thickest section and it becomes thinner while extending laterally^(5)^.

The pectineal ligament is primarily found on the lateral part of the prevesical and paravaginal space, forming the posterior border of the femoral canal, and has close proximity to the external iliac vessels, which lie on the superolateral part of the pectineal ligament. On the other hand, the pubic vein or arterial anastomosis between the inferior epigastric artery and obturator artery (corona mortis) is also adjacent to the pectineal ligament. At the inferolateral part of the pectineal ligament, the obturator nerve and obturator vessels are found. This obturator region consists of many vascular variations and anastomoses that the surgeon should be careful of during operations regarding the iliopectineal line and pectineal ligament.

## Materials and Methods

**Figure 1:**
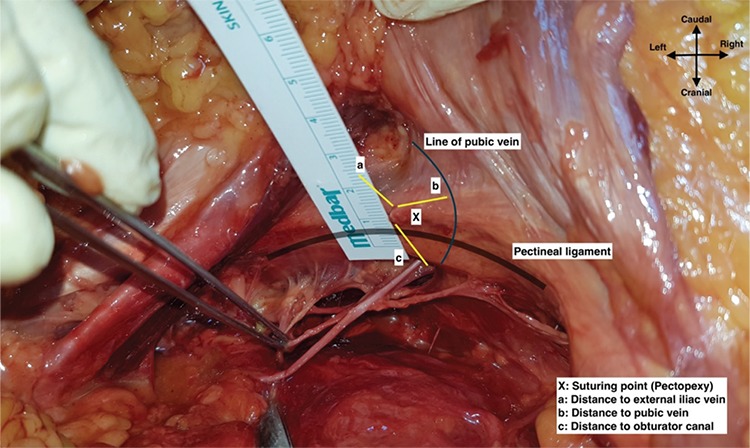
How the measurements were performed and the distance between the lateral portion of the middle of pectineal ligament and vascular structures, left pelvic side wall (here, the anterior abdomen was retracted excessively to take a clear photo, measurements were performed in the classic anatomic position as in surgery)

This study did not involve any cadaver of or tissue from the recently dead, other than bequeathed cadavers and tissue obtained in the normal course of necropsy, and therefore did not require ethical board approval and informed consent. All the cadavers were paid the greatest respect during the dissection for educational purposes. A total of seven fresh frozen female cadavers were dissected. A full pelvic dissection was performed to obtain a clear vision of the anatomic landmarks, at the anatomy laboratory of Bahcesehir University Department of Anatomy. The dissections and measurements of the anatomic structures were performed by senior surgeons and anatomists (E.Ç., O.D., I.S.). The distance between the midpoint of the pectineal ligament, which is the surgical suturing area during pectopexy surgery, and the external iliac vein, pubic anastomotic vessel (corona mortis) and obturator canal was measured bilaterally (Figure 1 demonstrates how the measurements were performed and the distance between the lateral portion of the middle of pectineal ligament and vascular structures). Statistical analyses were performed using a standardized computer-based calculating system. Descriptive analysis was made and reported as mean ± standard deviation (SD).

### Technique of cadaveric dissection

A midline vertical incision extending from the xiphoid process to the pubic symphysis circumferential around the umbilicus was performed to enter the abdominal cavity. The second and third incision were performed bilaterally following the costal margin from the xiphoid process to the midaxillary line and from the pubic symphysis to the anterior superior iliac spine, respectively, to maintain a full exposure of the abdominal cavity. The lateral parietal peritoneum covering the pelvic side wall was cut 2 cm cranial to the round ligament of the uterus and the retroperitoneal area was accessed. The psoas major muscle was exposed and on the medial part of the psoas major muscle the reflection of external iliac vessels was identified, attached to the psoas major muscle. The anterior and posterior leaf of the broad ligament was cut caudally and cranially, respectively, by preserving the round ligament of uterus. The paravesical space lateral to the bladder was developed and the obliterated umbilical artery was found and secured. The lateral part of the obliterated umbilical artery was identified in the obturator space. The median umbilical ligament was identified and cut at the level of pubic symphysis, the peritoneum covering the urinary bladder was reflected posteriorly and a blunt dissection was performed between the urinary bladder and the pubic symphysis to develop the prevesical space (retropubic space, Retzius space).

**Figure 2:**
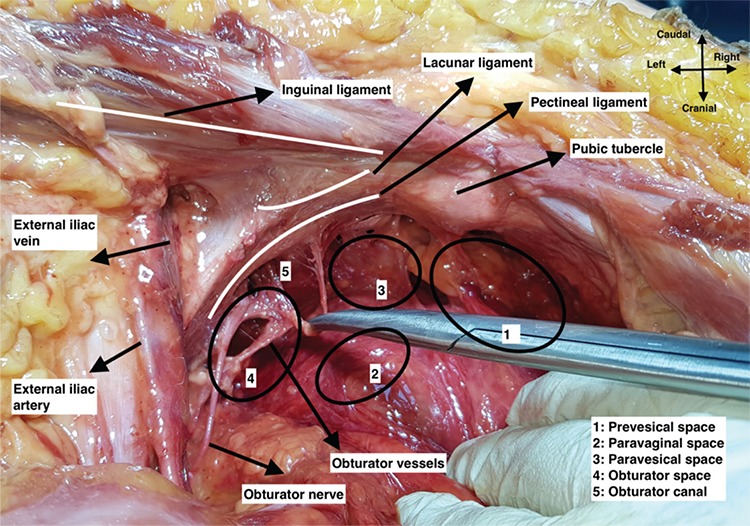
Pelvic anatomy and anatomic landmarks in a cadaver, left pelvic side wall

Afterwards, all fatty-lymphatic tissue covering the external iliac vessels and obturator fossa was removed with a meticulous dissection to obtain a straight measurement. Following the pathway of round ligament to the abdominal wall, the inguinal canal was identified and the inguinal ligament was exposed at the base of the rectus abdominis muscle. At this point, the inferior epigastric vessels were dissected and secured. On the medial part of the inguinal ligament, the aponeurotic expansion to the pectineal line is called the lacunar ligament. Here, the pubic anastomotic vessels known as corona mortis were dissected and secured. Antero-medial to the lacunar ligament, the pubic tubercle was palpated, and the laterally extending ligamentous structure over the periosteum of the pectineal line, which is called the pectineal ligament, was identified. The origin of the pectineal ligament was defined as its attachment point to the pubic tubercle. Figure 2 demonstrates the pelvic anatomy and anatomic landmarks, after cadaveric dissection.

## Results

**Figure 3:**
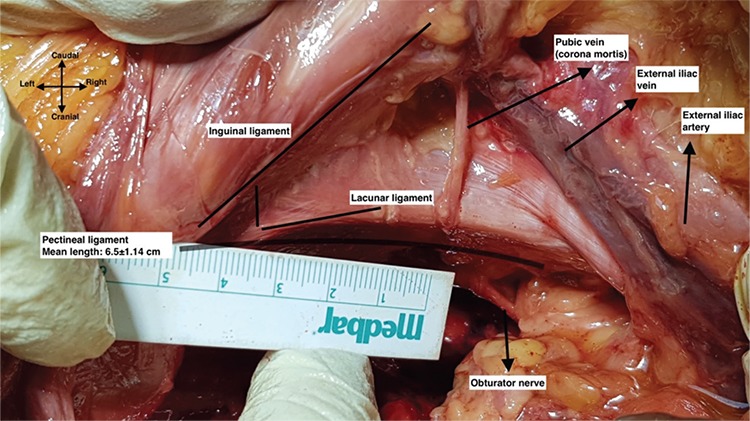
Anatomy and the mean length of pectineal ligament, right pelvic side wall

The mean age of the cadavers was 74±10.32 (range, 58-88) years. The mean Body Mass index (BMI) of the cadavers was 22.14±4.31 kg/m^2^ (Table 1). None of the cadavers had a history of pelvic surgery. After the origin of the pectineal ligament was determined, the measurements were obtained (Table 2). The total length of the pectineal ligament was 5.9±0.76 cm on the left and 6.5±1.14 cm on the right side; the midpoint of the pectineal ligament was 2.8±0.52 cm on the left and 3.6±0.47 cm on the right side. Figure 3 demonstrates the mean length of pectineal ligament. From the midpoint of the left pectineal ligament, the mean distance to the left external iliac vein was 1.04±0.23 cm, the mean distance to the left corona mortis was 2.15±0.48 cm, and the mean distance to the left obturator canal was 3.12±0.95 cm. From the midpoint of the right pectineal ligament, the mean distance to the right external iliac vein was 1.25±0.43 cm, the mean distance to the right corona mortis was 2.37±0.63 cm, and the mean distance to the right obturator canal was 3.57±0.93 cm.

## Discussion

Surgical anatomy of the pectineal ligament is especially important for urogynecology practice regarding POP and SUI operations. The anatomic location of the pectineal ligament may lead to some complications during surgery. In this respect, knowing the proximity of relevant anatomic landmarks to the pectineal ligament may improve the surgical outcomes and decrease probable complications. In this study, the surgical dissection of seven cadavers revealed that the closest anatomic structure to the pectineal ligament was the external iliac vein on both sides of the pelvis; 1.04±0.23 cm and 1.25±0.43 cm on the left and right side of pelvis, respectively. Additionally, the pubic vascular anastomosis called the corona mortis was also quite close to the surgical anchoring point; 2.15±0.48 cm and 2.37±0.63 cm on the left and right side of pelvis, respectively. However, the obturator canal had no close relation with the pectineal ligament; 3.12±0.95 cm and 3.57±0.93 cm on the left and right side of pelvis, respectively.

There is a controversy on the origin and texture of the pectineal ligament because the anatomy of the inguinal region has a complex architecture with regard to the contributing ligaments and attachments. The debate on the pectineal ligament may be considered as an option for further studies; however, in the literature, it has been shown that the aponeurosis and fascia of pectineus muscle, attachments of the inguinal ligament and iliopubic tract, transversalis fascia, and fibers from the psoas minor muscle and periosteum over the pectineal line all appeared to be a part of the pectineal ligament^(6,7,8,9,10)^. Rouviere^(11)^ stated that the pectineal ligament was the thickening of the fascia of the pectineus muscle, and therefore it had a strong structure. Additionally, the anatomic and histologic findings of Faure et al.^(1)^ in a cadaveric dissection study also showed that the pectineal ligament was the thickening of the pectineus fascia, rather than thickening of the periosteum.

On the other hand, classically, it is known that the pectineal ligament lies from the pubic tubercle to the iliopubic eminence, attaches to the periosteum of the superior pubic ramus, and covers the pectineal line of the pubis while extending dorsally. In this study, we measured the total length of pectineal ligament as 5.9±0.76 cm and 6.5±0.1.14 cm on the left and right side, respectively, and the midpoint of the pectineal ligament was 2.8±0.52 cm and 3.6±0.47 cm far from the pubic tubercle on the left and right side, respectively. Faure et al.^(1)^ found similar results that the average total length was 53 (range, 25-65) mm, and the midpoint of the pectineal ligament was 3-4 cm away from the medial insertion. On the far lateral portion, the pectineal ligament becomes thinner and fuses with the transversalis fascia at the part of iliopubic eminence; however, the medial portion of the pectineal line, which corresponds to the midpoint of pectineal ligament, just below the lacunar ligament, constitutes a strong band^(12)^. Due to the remarkable strength of the pectineal ligament, its importance has been emphasized in many surgical studies.

Pectopecxy surgery was suggested as a novel technique in POP surgery. During pectopexy surgery, the lateral portion of the middle part of pectineal ligament is used as the suturing point; however, it is well known that the inferomedial part of the pectineal ligament is used in Burch’s colposuspension procedures, which are performed for SUI^(2,13)^. Topographic anatomy yields that the risk of gross vessel injury may increase by proceeding towards the lateral part in pectopexy surgery, and our study demonstrated the closest anatomic structure was the external iliac vein.

Sacrocolpopexy and sacrouteropexy procedures are commonly performed for apical POP, in which the prolapsed cervix or vaginal vault is suspended to the sacrum with a mesh. Performing this procedure needs a great deal of experience and it represents a high learning curve. Because of the close anatomical structures which are present at the operation field; small intestines, sigmoid colon, ureter and presacral vessels are in risk of injury. Kale et al.^(14)^ conducted a case series study and applied pectopexy to seven patients with apical prolapse and reported no intraoperative and postoperative complications. During the pectopexy surgery, the pectineal ligament represents a distant space from the intestines, sigmoid colon, presacral vessels, and ureter, thus they stated that the pectineal ligament maintains a safe surgical field in pectopexy surgery. Moreover, Kale et al.^(14)^ offered this technique as a feasible procedure because the surgeon uses a wide area in the pelvis and the strong nature of pectineal ligament would decrease the postoperative recurrence rates. Our study did not demonstrate the exact anatomic distance to these structures; however, our anatomic dissections revealed that the small intestines and sigmoid colon were far away from the operation field unless an adhesion was detected. Presacral vessels are not in the surgical field and medial traction of the posterior leaf of the broad ligament will separate the ureter from the surgical area, so the risk of injury to these anatomic structures is less than with sacrocolpopexy procedures.

Noe et al.^(15)^ analyzed the results of the pectopexy and sacrocolpopexy procedures that were performed to 43 and 40 patients, respectively; they found that the duration of surgery and intraoperative blood loss was lower in the pectopexy group. Also, there were no severe intraoperative complications in either group such as bleeding, neural, vascular or intestinal injury. Banerjee and Noé^(2)^ compared 12 pectopexy cases with 242 sacrocolpopexy cases, there was no severe bleeding, neural, vascular or intestinal injury in the pectopexy group; however, the rate of bladder and intestinal injury was 0.7% and 1.2%, respectively, in the sacrocolpopexy group. Joshi et al.^(16)^ retrospectively evaluated consecutive pectineal ligament hysteropexy procedures. The results of 176 open and 18 laparoscopic cases demonstrated no intraoperative complications. In this respect, pectopexy surgery has a decreased risk of injury compared with major abdomino-pelvic organs; however, the risk of external iliac vein injury is increased for pectopexy procedures because of the close proximity. Despite this anatomic relevance, no injury has been reported in the literature for pectopexy surgery.

The anastomotic vessel between the external iliac and obturator vessels is called the pubic vein or artery, historically known as the corona mortis, which represents a danger zone, especially during femoral hernia repair operation. Nevertheless, when the retroperitoneum is accessed through the pelvic cavity, the risk of injury to the corona mortis is not frequent and is easily controlled^(17)^. The pubic anastomotic vessel is mostly detected as a vein and it lies behind the superior pubic ramus. The distance from the pubic symphysis varies between 40 and 96 mm^(18)^. We found the distance between the pectineal ligament and the corona mortis as 2.15±0.48 cm and 2.37±0.63 cm, on the left and right side, respectively. Surgeons should be careful not to injure the corona mortis, which is close to the pectineal ligament suturing point.

The obturator neuro-vascular bundle passes through the obturator canal and our study also demonstrated the distance of pectineal ligament to the obturator canal. The obturator artery and vein mostly lie posterior to the obturator nerve in the deep pelvic side wall within the fatty-lymphoid tissue. Previously, the length between the middle of the ichiopubic ramus to the obturator canal was analyzed and the researchers found the distance as 4.4 cm; this is especially important during retro-pubic anti-incontinence surgical procedures ^(19,20)^. Nonetheless, surgeons must know the limitations and risks of new procedures they are going to perform. The distance of the midpoint of the pectineal ligament to the obturator canal was found as 3.12±0.95 cm and 3.57±0.93 cm, on the left and right side, respectively. From this point, the pectineal ligament is not close to the obturator canal, which is in the obturator space and surrounded by a fatty-lymphoid tissue.

In the English literature, we found no studies measuring the distance between the pectineal ligament, corona mortis, and other relevant vascular structures. In this respect, this study may make an important contribution to the current literature about apical prolapse surgery. The postmortem vasoconstriction and shrinkage of tissues will change the exact distance of surgical planes, but we used fresh frozen cadavers to optimize the measurements and decrease the lacking points in defining tissue planes.

As a conclusion, the pectineal ligament has a close relation with the main vessels in the pelvis. Indeed, positioning the suture to the lateral part of the midpoint of pectineal ligament increases the proximity with the external iliac vein, which was measured as the closest anatomic structure to the pectineal ligament in terms of pectopexy procedures. Pelvic surgeons must be careful and gentle, and retract the external iliac vein from the suturing point of pectineal ligament while performing pectopexy procedures.

## Figures and Tables

**Table 1:**
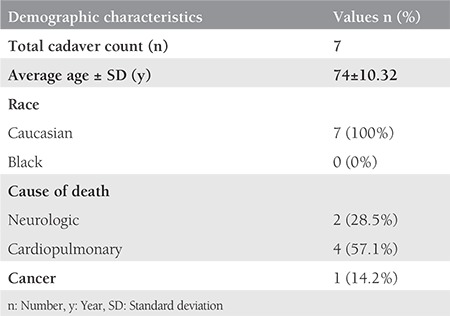
The demographic data of the cadavers

**Table 2:**
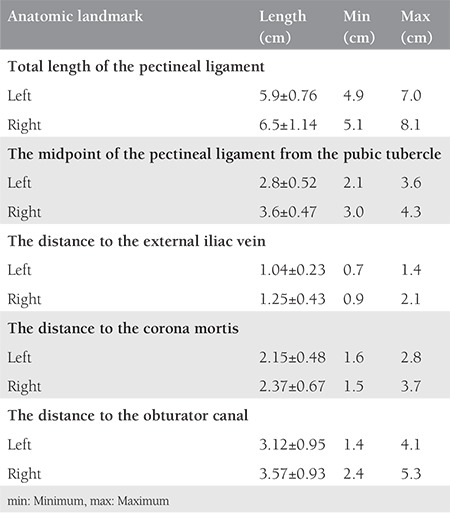
The distance of the pectineal ligament to the relevant anatomic landmarks
